# Biaryl Anion Radical Formation by Potassium Metal Reduction of Aryl Isocyanates and Triaryl Isocyanurates

**DOI:** 10.1021/acs.joc.4c01844

**Published:** 2024-10-13

**Authors:** Steven J. Peters, Sean H. Kennedy, Colton J. Christiansen

**Affiliations:** Department of Chemistry, Illinois State University, Normal, Illinois 61790-4160, United States

## Abstract

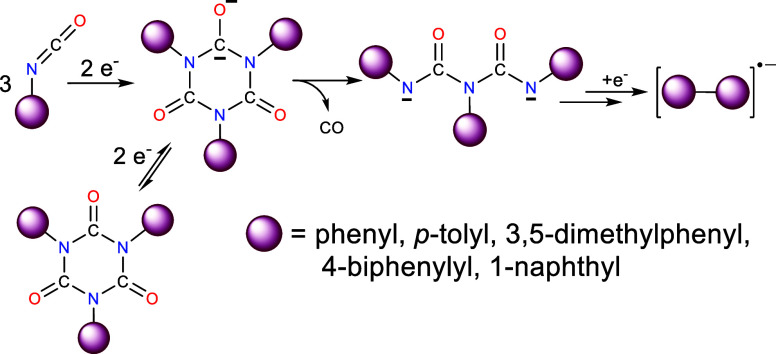

The potassium metal reduction of aryl isocyanates (aryl = phenyl, *p*-tolyl, 3,5-dimethylphenyl, 4-biphenylyl, and 1-naphthyl) in THF with 18-crown-6 or in HMPA results in the formation of the corresponding triaryl isocyanurate anion radicals. Continued exposure to potassium results in loss of the isocyanurate anion radical and the eventual formation of the respective biaryl anion radical. The 1,1′-binaphthyl anion radical is found to undergo a cyclodehydrogenation reaction, which leads to formation of the perylene anion radical. When authentic triaryl isocyanurates are reduced with metal, the heterocyclic ring undergoes fragmentation with elimination of carbon monoxide to produce a triarylbiuret dianion. This ring opening reaction is initiated by the two-electron reduction of the neutral isocyanurate species. The biaryl anion radical is formed when the biuret dianion is reduced further with metal. A possible mechanism for biaryl formation involves a heterolytic cleavage of an aryl C–N bond and release of an aryl radical once the triarylbiuret dianion is further reduced. A subsequent intermolecular reaction between two aryl radicals forms the corresponding biaryl, which can then be reduced to the anion radical. Notably, when a mixture of two different triaryl isocyanurate compounds is reduced in solution, the corresponding mixed biaryl anion radical is generated.

## Introduction

The versatility of isocyanates in synthetic chemistry has been recognized for almost two centuries, especially in the production of polymeric materials such as polyurethanes. Aryl isocyanates are used in the production of triaryl isocyanurates (1,3,5-triazine-2,4,6-triones), where these heterocycles are found to enhance the physical properties of polyurethanes by making them more transparent and chemically resistant, which has led to their commercial importance.^1−5^ These physical properties are due in part to the aromatic stability of the isocyanurate ring since the three nitrogen lone pairs contribute to the π conjugation within the ring. In addition, mesoporous organosilicas that include bridging isocyanurates are effective materials for removal of heavy metals in aqueous solutions and are found to be potential materials for carbon dioxide capture.^6−8^ Furthermore, low-toxicity isocyanurate siderophores have been investigated as candidates for antifungal agents and drug delivery in mammals.^9,10^ As the uses for isocyanurates continue to grow, more efficient synthetic methods for their synthesis from isocyanates have also continued.^11−16^

Isocyanates are also commonly used in the production of polymeric nylons, such as 1-nylon, which generate polyamide linkages that mimic extended helical secondary structure much like proteins. Synthesis of these nylon polymers includes the dimerization of isocyanate anion radicals to afford an oxanilide dianion, which initiates polymerization and the polyamide backbone formation.^17^ Synthetic 1-nylons are found to exhibit interesting physical and chemical properties and have possible uses in liquid crystalline materials as part of optical and molecular switches.^17−21^

There are numerous examples in the literature of single and multiple electron reductions involving arene systems that promote fragmentation of the reduced species via bond breakage followed by biaryl formation and, for many of these systems, the biaryl anion radicals are observed.^22−26^ Much of the interest in exploring biaryl formation comes from their use in functional materials, in natural products and as ligands in asymmetric catalysis.^27^ Past studies on the single electron reduction of *N*,*N*′-dimethyl *N*,*N*′-diarylureas have shown that biaryl anion radicals are formed via an intramolecular reductive elimination, reaction 1.^22^ The dimethyldiaziridione product also formed in the elimination reaction is believed to undergo polymerization under these reductive conditions.^22^ Multiple electron reduction reactions have also been shown to undergo heterolytic bond cleavage and the formation of biaryl anion radicals. Arenes that exhibit this reaction pathway include dinaphthyl ether, triphenyl amine, diphenyl silane, and trinaphthyl borane, to name a few.^23−26^Reaction 2 is an example that shows a heterolytic bond cleavage of triphenyl amine upon reduction with two electrons using alkali metals. Bond cleavage releases an aryl anion, which then reacts with the remaining diphenyl amine anion to produce biphenyl, which is then reduced to the anion radical.^26^
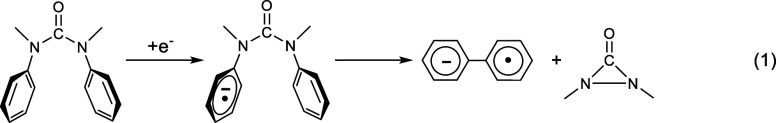
1
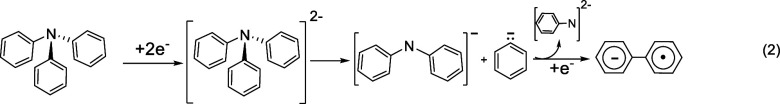
2

Previously, we reported that the product formed from the one-electron reduction of alkyl and aryl isocyanates (alkyl = Et and cyclohexyl and aryl = phenyl and 1-naphthyl) with alkali metals reacts rapidly with two additional neutral isocyanates to form the respective trialkyl and triaryl isocyanurate anion radicals.^28−30^ We determined that the majority of electron spin density is localized within the π system of a single carbonyl group within these isocyanurate anion radicals.^29^ Ab initio calculations revealed that the ring distorts around this carbonyl carbon, altering the geometry from trigonal planar to trigonal pyramidal with significant elongation of the two carbon–nitrogen amide bonds associated with the region of high electron density.^28,29^ No one has looked at the multiple electron reduction of triaryl isocyanurates and whether the addition of more than one electron to the π system of the isocyanurate will cause instability in the ring due to the increase in charge buildup within a carbonyl moiety. This instability may result in the isocyanurate ring undergoing fragmentation and the formation of new anion radical species, as was observed with other arene systems described above.

Since our previous studies have shown that triaryl isocyanurate anion radicals can be formed in situ by the one-electron reduction of the aryl isocyanate monomers,^28−30^ we were motivated to explore how additional exposure to potassium metal will affect the stability of the isocyanurate ring and, moreover, the chemistry that occurs upon multiple electron reductions. In the studies presented here, we find that multiple electron reduction leads to fragmentation of the isocyanurate ring and formation of biaryl anion radicals.

## Results and Discussion

### Aryl Isocyanate Reductions with Excess Metal

Potassium metal reduction experiments were carried out either in tetrahydrofuran (THF) with a molar excess of 18-crown-6 (relative to the aryl isocyanate and triaryl isocyanurate) or in hexamethylphosphoramide (HMPA). These experimental conditions help to facilitate the release of electrons (e.g., solvated electrons) as the solution comes into contact with the potassium metal surface^31−33^ and have been used in the reduction of other aryl systems.^34,35^ Notably, potassium is found to be a strong reducing agent in HMPA when compared with other single electron reductants.^36,37^ Exposure to the metal is carried out in a controlled manner, which means the reduction process can be performed gradually as the solution is exposed to more and more metal (see Experimental Section).

**Figure 1:**
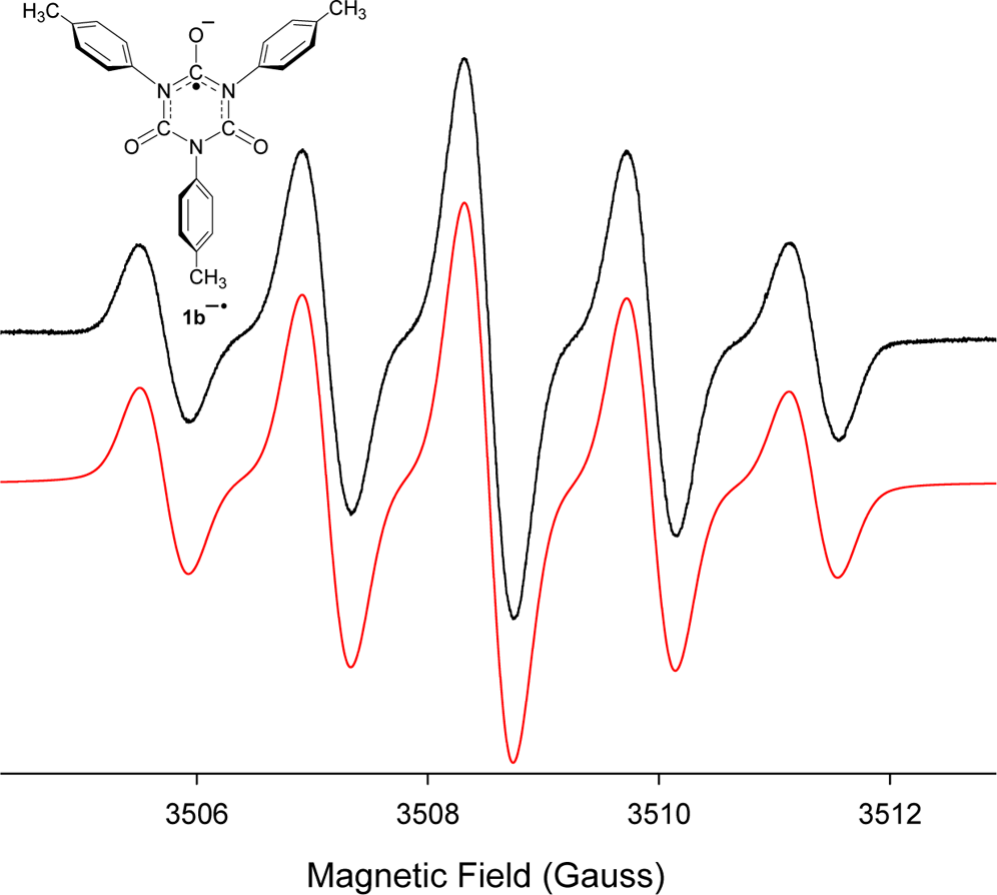
(Black) EPR spectrum recorded at 295 K after a THF solution containing *para*-tolyl isocyanate and 18-crown-6 was exposed to a *K* metal mirror under vacuum. (Red) Computer-generated simulation using a_N_ of 1.40 G (2 ^14^N atoms). Small unresolved hyperfine coupling from two tolyl groups was necessary to improve the fit of the simulation and are a_H_ of 0.10 G (4H atoms) associated with the *ortho* hydrogens and a_H_ of 0.11 G (6H atoms) from the two *para* methyl groups. The Δ*w*_pp_ = 0.21 G.

When a THF solution containing *p*-tolyl isocyanate (**1a**) and two equivalents of 18-crown-6 is initially exposed to potassium metal (i.e., [*K*] < [**1a**]), a light orange color is produced, and the resulting solution exhibits a strong EPR signal upon analysis, Figure 1. The spectrum shows hyperfine coupling between the unpaired electron and two equivalent nitrogen atoms and additional unresolved coupling to hydrogens associated with the two tolyl rings attached, which accounts for the broadness in the observed pentet, Figure 1 (note that hyperfine coupling to the third nitrogen is small (*a*_N_ < 0.09 G) and lost in the line width^29^). These results match those of previous studies by us where the reduction of phenyl isocyanate produces the anion radical of triphenyl isocyanurate.^29^ As with both of these systems, the electron is localized in one of the carbonyl moieties with most of the electron spin density residing on the *sp*^2^ hybridized carbon causing a Jahn–Teller distortion, resulting in loss of planarity within the reduced isocyanurate ring.^28,29^ Therefore, the initial exposure of **1a** to metal generates the tri*p*-tolyl isocyanurate anion radical (**1b**^•–^) formed by a rapid cyclotrimerization between **1a**^•–^ and two additional **1a** molecules, reaction 3. Encapsulation of the K^+^ ion by the crown ether along with the steric effects associated with the two *p*-tolyl moieties make formation of a tight ion pair between **1b**^•–^ and the cation difficult; however, loose or solvent separated ion pairs likely still persist in solution.^29^
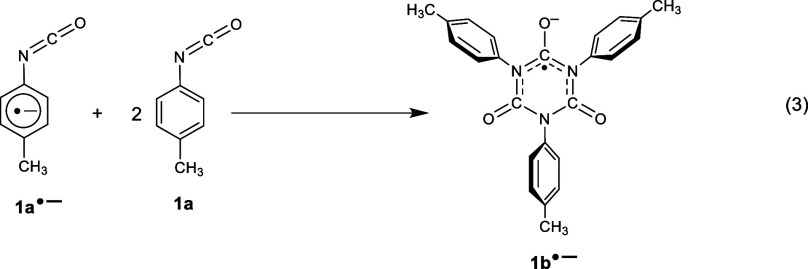
3

**Figure 2:**
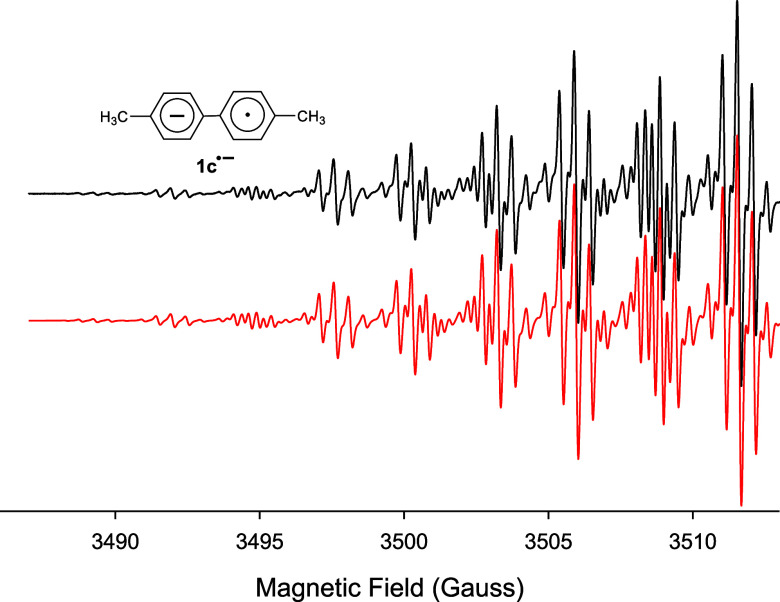
(Black) Downfield half of the EPR spectrum recorded at 295 K after addition of excess *K* metal to a THF solution containing *para*-tolyl isocyanate (**1a**) and 18-crown-6 under vacuum. (Red) Computer-generated simulation using a_H_’s of 0.51 and 2.68 G for two sets of 4H atoms and a_H_ of 5.65 G for six H atoms associated with coupling to the two methyl groups, Δ*w*_pp_ = 0.15 G.

Additional exposure of this THF/18-crown-6 solution to potassium metal causes the EPR signal for **1b**^•–^ to gradually disappear. As the metal reduction is continued, after **1b**^•–^ is no longer observed, the orange color of the solution darkens considerably and no new paramagnetic species is detected. These solution colors are displayed in Figure S1 in Supporting Information with the first three samples, starting from the left, showing the extent of the increased reduction with potassium metal. Finally, when the solution has been exposed to a large excess of metal (i.e., [*K*] ≫ [**1b**^•–^]), the solution color changes rapidly from orange to dark brown (see the fourth sample from the left in Figure S1), and a new anion radical is detected that exhibits a strong, well-resolved EPR signal. The computer simulation of this EPR spectrum indicates that the bi*p*-tolyl anion radical (**1c**^•–^) is formed, as shown in Figure 2. Interestingly, the brown color of the solution may result from the blue color, which is indicative of the presence of biaryl anion radicals in solution, mixing with the orange color observed prior to the formation of **1c**^•–^.

We have also performed potassium metal reductions with phenyl isocyanate (**2a**) and 3,5-dimethylphenyl isocyanate (**3a**) under the same THF/18-crown-6 conditions, and these two isocyanates give the analogous results obtained from the reduction of **1a**, where the initial exposure to metal generates the triaryl isocyanurate anion radicals, **2b**^•–^ and **3b**^•–^, respectively. As the reduction with *K* metal continues, the EPR signal for these isocyanurate anion radicals disappears, and after a significant amount of metal has been added, we obtain strong, well-resolved EPR spectra for the biphenyl anion radical (**2c**^•–^) and for the 3, 3′, 5, 5′-tetramethyl-biphenyl anion radical (**3c**^•–^); the EPR spectra for both are displayed in Figures S2 and S3, respectively. We also performed potassium metal reduction in HMPA on all three aryl isocyanates, and the analogous biaryl anion radicals were formed.

**Figure 3:**
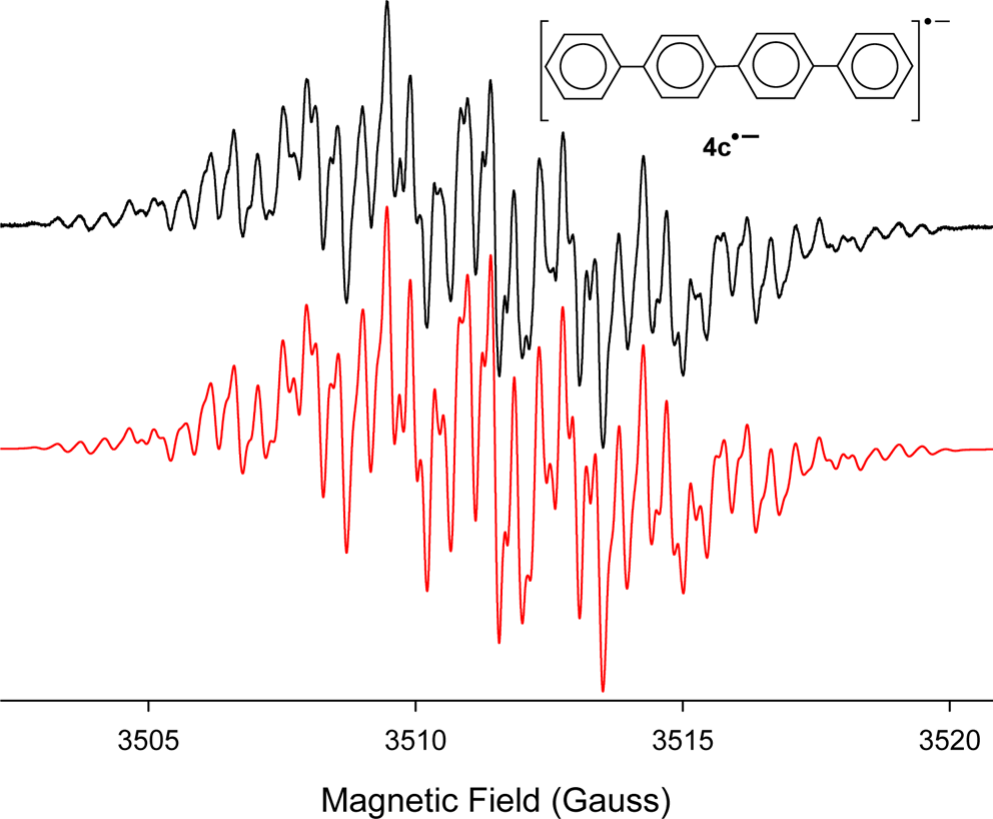
(Black) X-band EPR spectrum recorded at 295 K after addition of excess *K* metal to a THF solution containing 4,4′-biphenylyl isocyanate (**4a**) and 18-crown-6 under vacuum. (Red) Computer simulation using a_H_’s of 0.06, 0.43, 1.33, and 1.54 G for four sets of 4H atoms and an a_H_ of 1.92 G for a pair of H atoms, Δ*w*_pp_ = 0.12 G.

**Figure 4:**
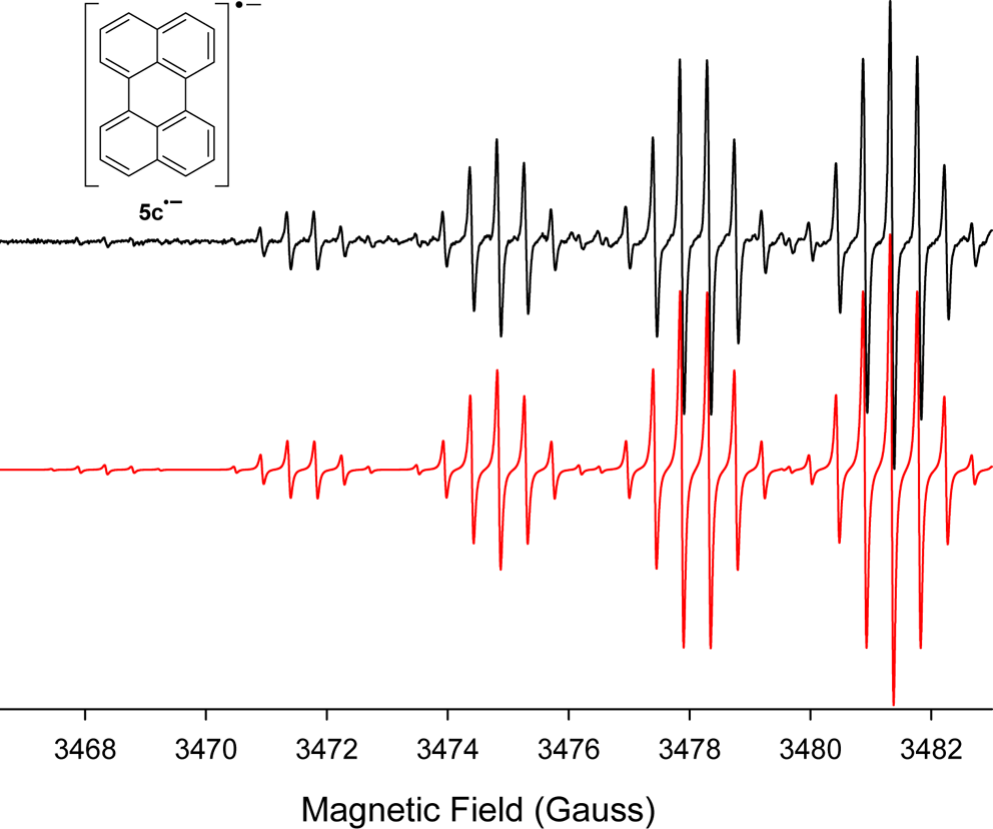
(Black) X-band EPR spectrum (downfield half) recorded at 295 K after addition of *K* metal to a HMPA solution containing 1-naphthyl isocyanate (**5a**) under vacuum ([*K*] ≫ [**5a**]). (Red) Computer simulation using a_H_’s of 0.44 G, 3.02 G, and 3.48 G for three sets of 4H atoms, Δ*w*_pp_ = 0.063 G.

**Figure 5:**
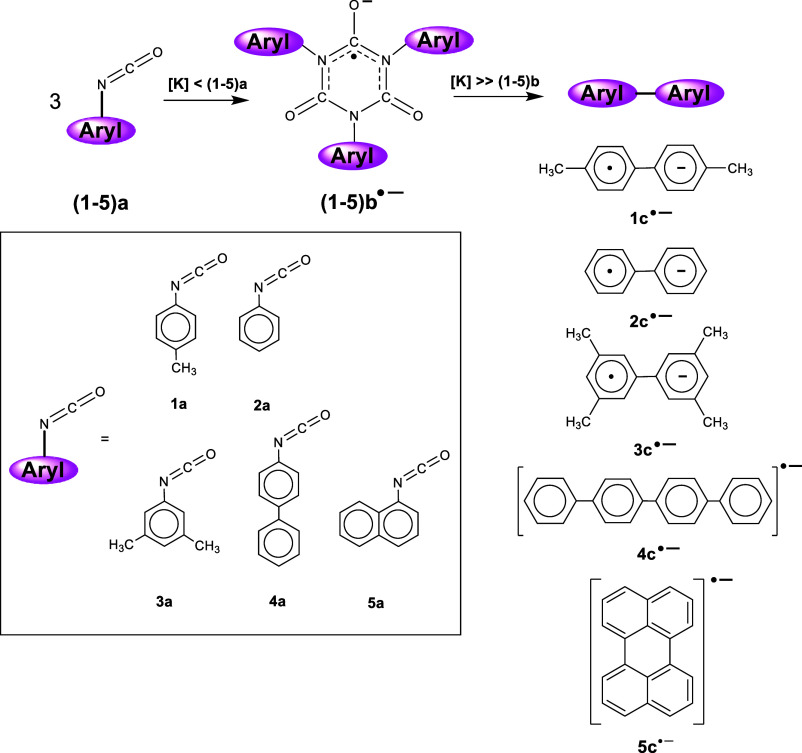
Reaction summarizing the formation of the biaryl anion radicals (**1c**^•–^– **4c**^•–^) and the perylene anion radical (**5c**^•–^) from the potassium metal reduction of the five respective aryl isocyanates (**1a**–**5a**) in THF with excess 18-crown-6 or in HMPA.

We find that isocyanates with larger aryl rings attached produce similar results when they are reduced under the same conditions. For example, after a significant amount of metal has been added to a solution containing 4,4′-biphenylyl isocyanate (**4a**), a strong well-resolved EPR spectrum for *p*–*p*’-quaterphenyl (or tetraphenyl) anion radical (**4c**^•–^) is obtained, Figure 3. Note that the coupling constants used in the computer simulation match the previously published values.^38^ Interestingly, when we performed the reduction of a solution containing 1-naphthyl isocyanate (**5a**), EPR analysis did not produce the expected signal for the 1,1′-binaphthyl anion radical. Instead, the spectrum for the perylene anion radical (**5c**^•–^) was obtained, as shown in Figure 4. Although this result was initially surprising, there are several examples in the literature that describe a pathway to perylene from the anion radical of 1,1-binaphtyl.^39−41^ Therefore, it is likely that the 1,1′-binaphthyl anion radical is formed in the reduction of **5a**, but quickly undergoes an anionic cyclodehydrogenation reaction under these highly reduced conditions to form **5c**^•–^, reaction 4.^39,40^Figure 5 is a compilation of the potassium metal reduction experiments performed on the five aryl isocyanates in these studies.
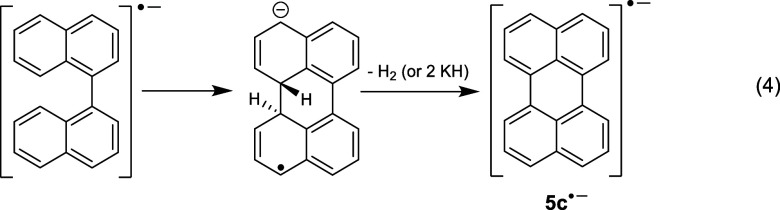
4

In reduction experiments with **1a** and **2a**, attempts were made to determine the approximate yield for both biaryl products formed. Once the strongest EPR signal was obtained with exposure to metal, solutions were quenched with excess iodine, resulting in the rapid oxidation of the anion radicals to their neutral state (e.g., **1c**^•–^ + 1/2 *I*_2_ → **1c** + *I*^–^). The apparatus was then opened, and the THF solution was quenched with an aqueous solution containing sodium thiosulfate (used to reduce the excess iodine), followed by extractions with ethyl acetate. The organic layer was analyzed by using GC coupled with flame ionization detection. An internal standard (triethyl isocyanurate) of known concentration was added to the ethyl acetate solution and used to quantify the amount of biaryl formed in these experiments. We find that the approximate yield was close to 10% for both biaryl products (i.e., bi*p*-tolyl and biphenyl) formed in these reduction experiments. Although relatively low, these yields are similar to that found in other studies involving the formation of biaryls using similar reduction techniques.^24,26^ However, further studies are underway to determine whether the yields can be improved under different reductive conditions.

### Authentic Triaryl Isocyanurate Reduction Experiments

The results described above suggest that formation of the biaryl anion radicals is possible from the direct reduction of the triaryl isocyanurates since these heterocycles were formed early in the reduction of the aryl isocyanates. To prove this, analogous reduction experiments were carried out on two triaryl isocyanurates [e.g., tri*p*-tolyl isocyanurate (**1b**) and triphenyl isocyanurate (**2b**)], which were synthesized in our laboratory using known procedures.^42^ The reduction of these pure independently synthesized isocyanurates was carried out using the same conditions as those used in the isocyanate experiments. When a solution containing either **1b** or **2b** was initially exposed to a deficient amount of metal, a weak EPR signal was observed for the respective triaryl isocyanurate anion radical (**1b**^•–^ or **2b**^•–^), which gradually disappeared as more metal was added. With continued exposure to metal, the orange color of each solution darkened considerably in the same manner as that for the aryl isocyanate reduction experiments. When enough metal was added to change the color of the solution to dark brown, strong EPR signals for both biaryl anion radicals (**1c**^•–^ or **2c**^•–^) were observed. Notably, the production of these biaryls must then involve a chemical change with the triaryl isocyanurates when they are reduced with enough electrons.

**Figure 6:**
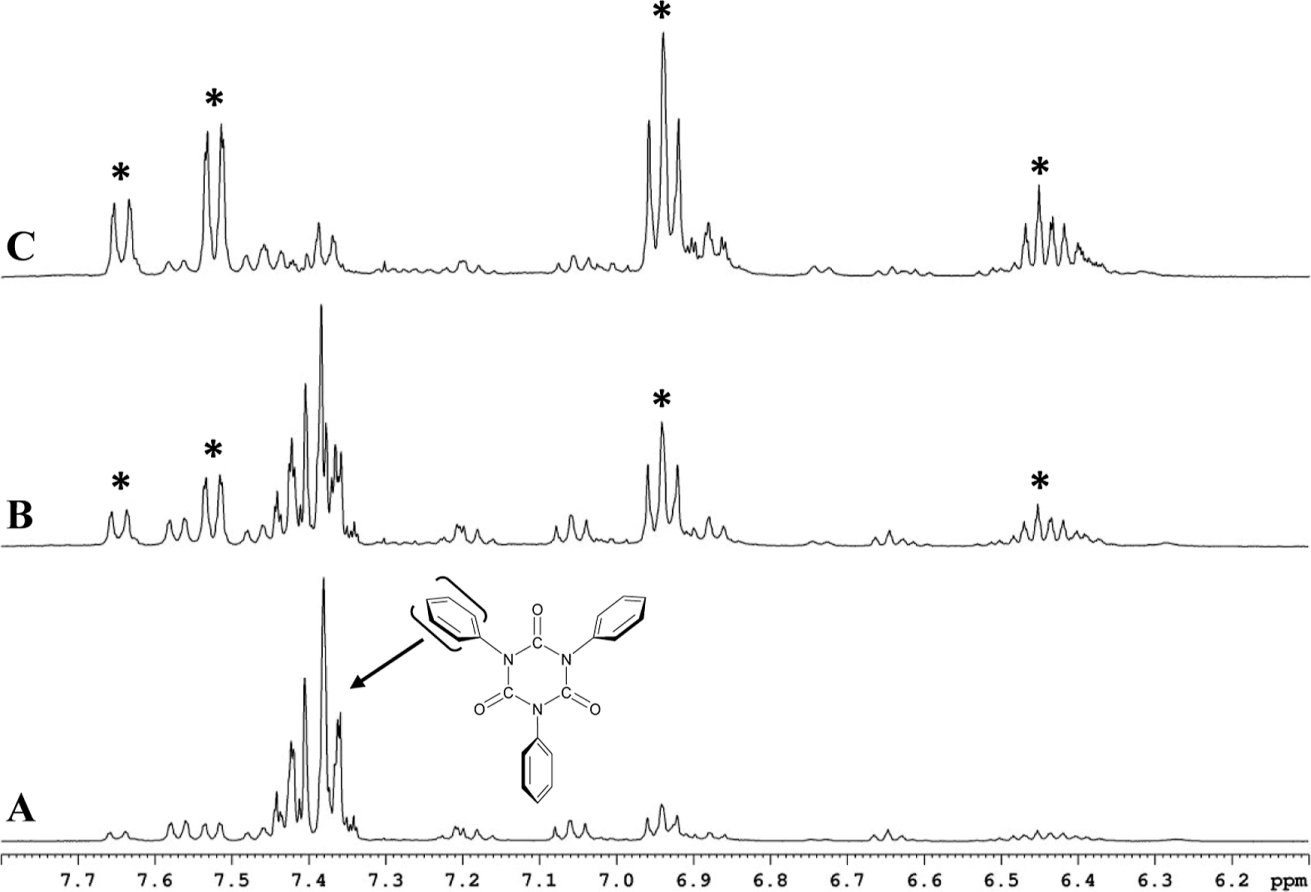
400 MHz ^1^H NMR spectra of a THF-*d*_8_ solution containing triphenyl isocyanurate (**2b**) with three equivalence of 18-crown-6 reduced sequentially with *K* metal under vacuum. The packet of resonances from 7.45 to 7.32 ppm are the three phenyl ring protons in **2b** (the chemical shift of 18-crown-6 is at 3.5 ppm.). (A) Deficient amount of metal used to reduce the solution where [*K*] < [**2b**]. (B) Further reduction of the solution with *K* metal where [*K*] ∼ [**2b**]. (C) Final exposure of the solution to *K* metal where [*K*] ∼ 2 × [**2b**]. Note that the asterisks mark the resonances associated with the dominant product formed in the reduction process.

**Figure 7:**
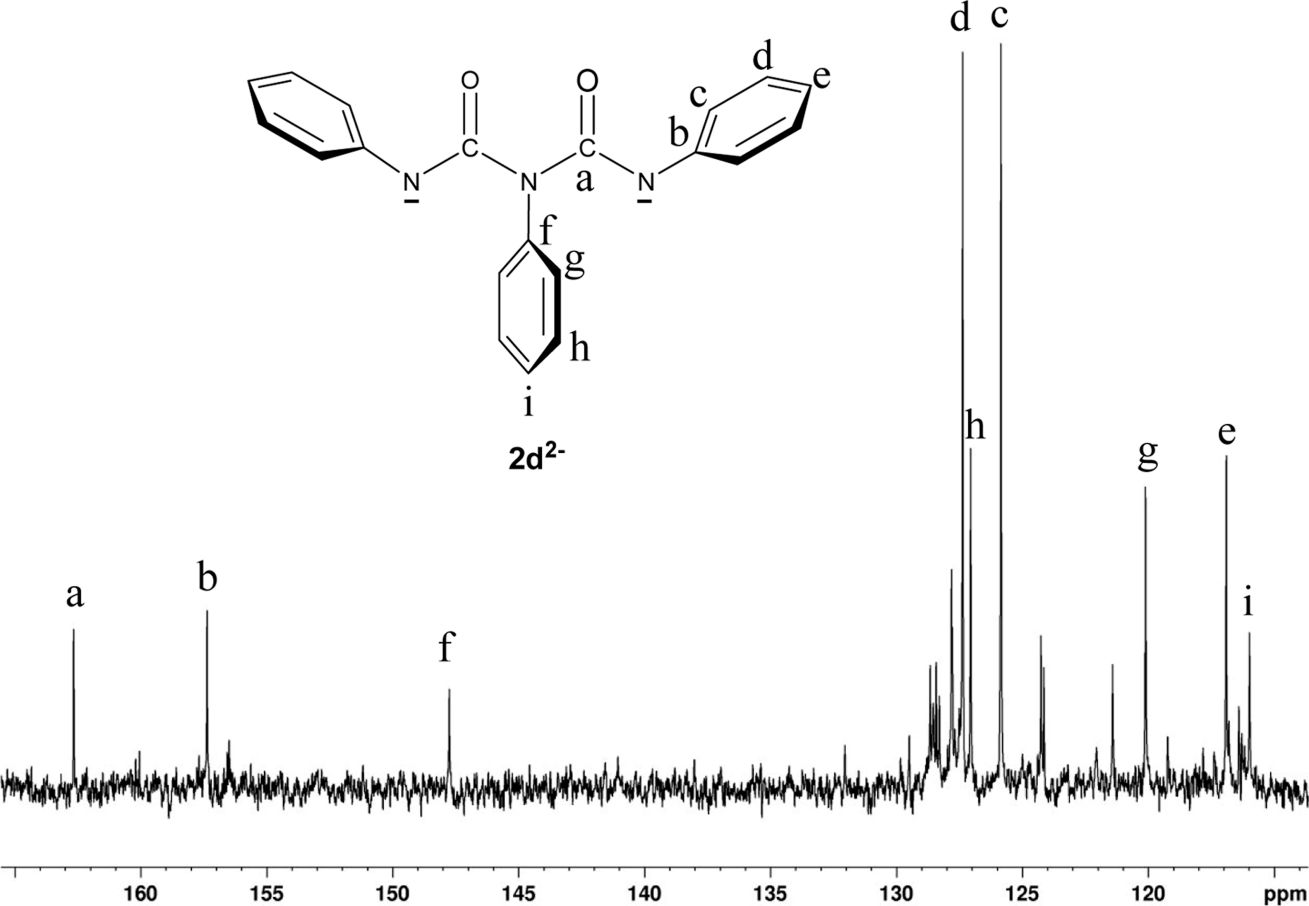
100 MHz ^13^C{^1^H}-NMR spectrum of a THF-*d*_8_ solution with triphenyl isocyanurate (**2b**) and three equivalence of 18-crown-6 reduced with *K* metal under vacuum. This spectrum was obtained for the third NMR sample and was collected once the solution was reduced with the largest amount of metal (i.e., where [*K*] ∼ 2 × [**2b**]).

To explore the changes that occur to the triaryl isocyanurates upon exposure to metal, we utilized NMR spectroscopy to investigate the reduction of **2b** in THF-*d*_8_ with excess 18-crown-6 using a glass apparatus equipped with three NMR tubes. Vacuum-sealed NMR samples were collected for analysis at three different times in the reduction with *K* metal. In these experiments, we used the color of the solution as an indicator for determining when to remove an NMR sample (examples of these colors are illustrated in Figure S1). Notably, the third and last NMR tube was collected before the solution turned brown, and before the biaryl anion radical formed in the reduction. Analysis of the three NMR samples revealed that fragmentation of the isocyanurate ring occurred throughout the reduction process. Figure 6 displays the ^1^H NMR spectra for the aromatic region associated with all three samples. The NMR spectrum for the first sample (Figure 6A) reveals that the isocyanurate ring has begun to breakdown after minimal exposure to metal. The strong multiplet between 7.45 and 7.32 ppm is assigned to the proton resonances associated with the three equivalent phenyl rings in **2b**. The weaker proton resonances observed reveal that **2b** has already begun to fragment this early in the reduction process. The spectrum shown in Figure 6B was collected on the second sample after the solution darkened considerably with the addition of more metal. We find that the fragmentation of **2b** has continued as more metal is added. A comparison of the spectra in Figure 6A,B shows that the resonances from **2b** have decreased in intensity by nearly a factor of 2 (based on integration), while the new phenyl resonances have increased in intensity (i.e., those marked with asterisks in Figure 6B) by approximately the same magnitude. We find that all the other weaker resonances found in the aromatic region are nearly static in both spectra. Finally, the NMR spectrum was collected for the third sample after significant reduction with metal ([*K*] ∼ 2 × [**2b**]), and the solution exhibited the darkest orange color (see Figure S1, third sample for reference) when compared with the other two NMR samples, Figure 6C. The resonances associated with **2b** are virtually gone, indicating that the breakdown is complete at this point in the reduction process. The resonances marked with asterisks have continued to grow and are now the dominant resonances in the spectrum. Integration of these new phenyl resonances in all three spectra reveals a constant 2:1 ratio of phenyl rings that suggests that this new product retains the three phenyl rings from **2b** but are clearly no longer equivalent. To help elucidate the structure of this fragmentation product, ^13^C-{^1^H}-NMR data (Figure 7) and 2D-NMR (COSY, HSQC and HMBC) data (Figures S4 and S5) were also collected for the third NMR sample. The ^13^C-{^1^H}-NMR spectrum exhibits strong resonances for a single carbonyl carbon at δ = 162.7 ppm and two ipso carbons at δ = 157.5 ppm and at δ = 147.8, which are associated with the two nonequivalent phenyl rings. The COSY ^1^H –^1^H ^3^J correlations (Figure S4) and the ^13^C–^1^H heteronuclear ^1^J_CH_, ^2^J_CH_, and ^3^J_CH_ correlations in the HSQC and HMBC spectra (overlaid in Figure S5) were used to make the ^1^H and ^13^C chemical shift assignments associated with the two nonequivalent rings.

The conclusion reached from these NMR experiments is that a reductive fragmentation or ring opening of the isocyanurate ring has taken place with exposure to the potassium metal and that the major product formed is the *N*, *N*′, *N*″-triphenyl biuret dianion (**2d**^2–^). Figure 7 gives the structure along with all of the ^13^C chemical shift assignments. In addition to the NMR results, we also performed density functional theory computations on **2d**^2–^ that include the predicted isotropic NMR shielding tensors for all carbons and hydrogens in **2d**^2–^.

**Figure 8:**
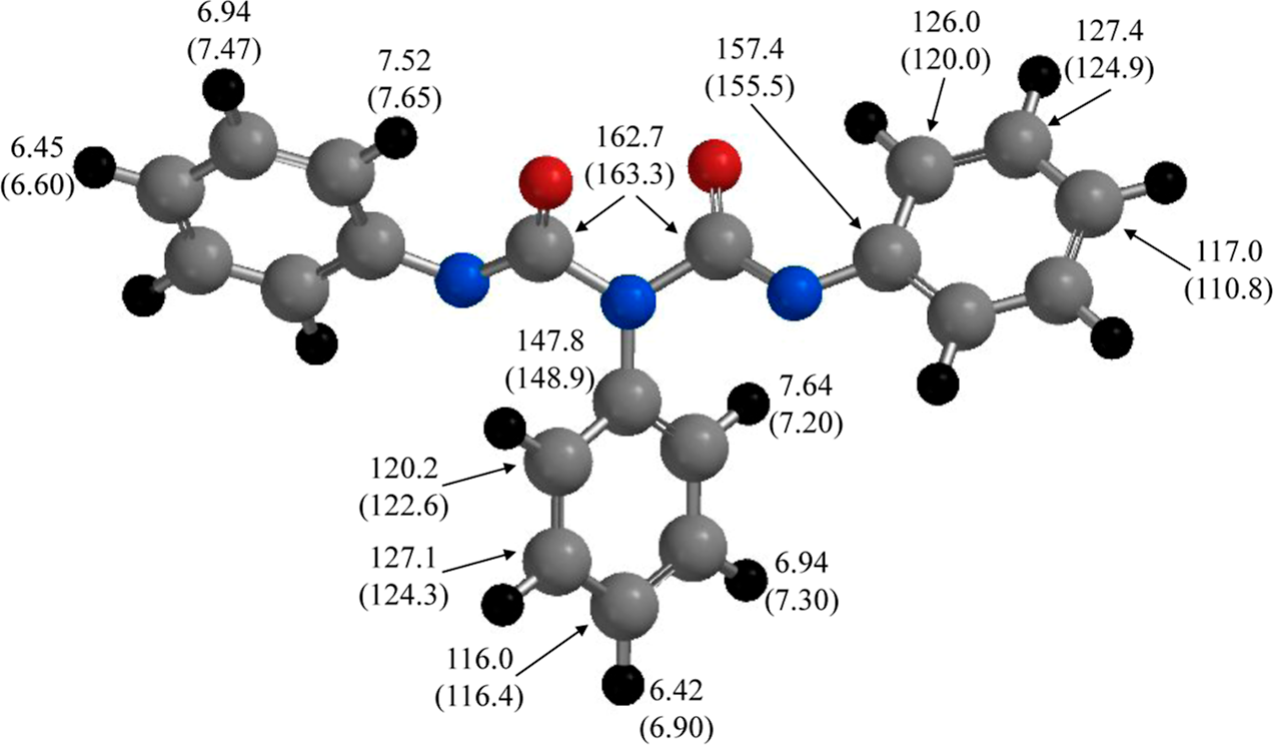
B3LYP/6-31+G(d,p) optimized structure for **2d**^2–^ showing experimental chemical shifts and calculated isotropic shielding tensors (in parentheses) and are given in ppm. The phenyl rings on N1 and N3 are equivalent; therefore, the hydrogen and carbon assignments shown apply to both rings. The calculated shielding tensors for TMS were used as a reference.

**Scheme 1:**
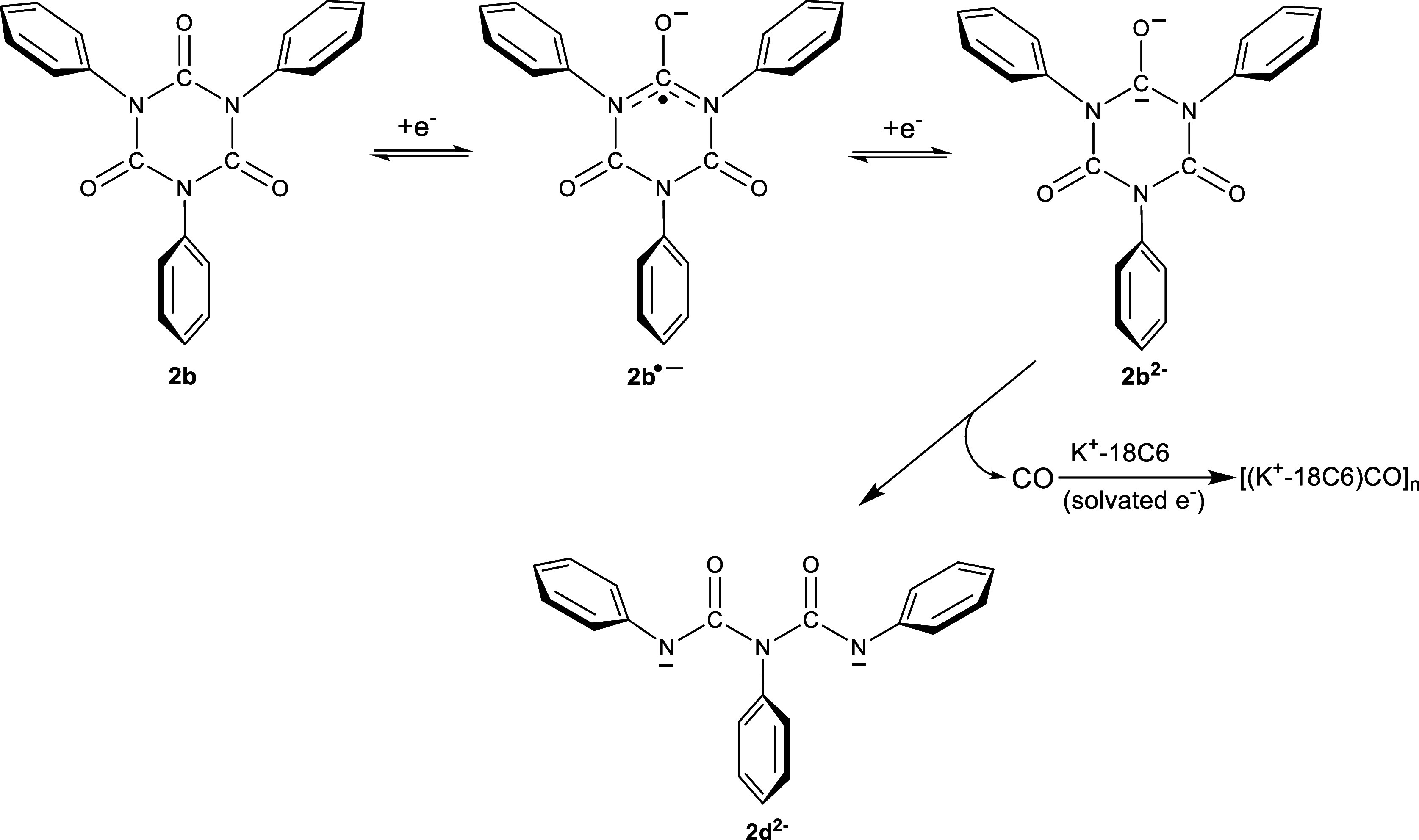
Pathway for the Multiple Electron Reduction of Triphenyl Isocyanurate (**2b**) Leading to the Formation of the N, *N*′,*N*″-triphenylbiuret Dianion (**2b**^2–^). A Similar Scheme Applies to the Other Triaryl Isocyanurate Systems (Aryl = *p*-Tolyl, 4′-Biphenylyl, 3,5-Dimethylphenyl, or 1′-Naphthyl) Reduced with Potassium Metal. (The 18-Crown-6-K^+^ Complex Is Shown Only for the Reduction of CO and Not with the Reduced Triaryl Species for Clarity)

The geometry for **2d**^2–^ was optimized using the B3LYP/6-31+G(d,p) protocol, and the same basis set was used to predict the isotropic NMR shielding tensors (in ppm), Figure 8. The optimization included the self-consistent reaction field with the polarizable continuum model (CPCM) to mimic the solvation properties of THF. The calculated shielding tensors were referenced to the isotropic shielding tensors for tetramethylsilane (TMS), also obtained via computations using the same level of theory (see Supporting Information for the NMR results on TMS). Remarkably, we find very good agreement between the isotropic shielding tensors and the experimental chemical shift for the carbonyl carbons and the two unique ipso carbons associated with the phenyl rings. Scheme 1 shows a proposed mechanism for the formation of **2d**^2–^ that involves the two-electron reduction of **2b** (e.g., **2b**^2–^), which then undergoes the elimination of CO to produce **2d**^2–^. The ^1^H and ^13^C NMR data (Figures 6 and 7) show no clear evidence of **2b**^2–^ in solution, which is further evidence of the instability in the isocyanurate ring when reduced with two electrons. The fate of CO after the fragmentation of **2b**^2–^ is not clear; however, there is evidence to suggest that the CO will undergo a reductive homologation reaction with solvated electrons and produce [(K^+^-18C6)CO]_*n*_ salts where n ∼ 2 to 6.^43,44^ Finally, the biuret backbone allows for greater charge separation in **2d**^2–^ when compared with that of the isocyanurate ring in **2b**^2–^, which may promote the ring opening reaction. Since all aryl isocyanates studied (e.g., **1a**–**5a**) generate triaryl isocyanurates (e.g., **1b**–**5b**) as anion radicals when initially exposed to metal, we expect that an analogous ring opening with elimination of CO will also produce the triarylbiuret dianions (e.g., **1d**^2–^ – **5d**^2–^) upon reduction with multiple electrons.

### Reduction of Triaryl Biuret Dianions and Formation of the Biaryl Anion Radicals

**Scheme 2:**
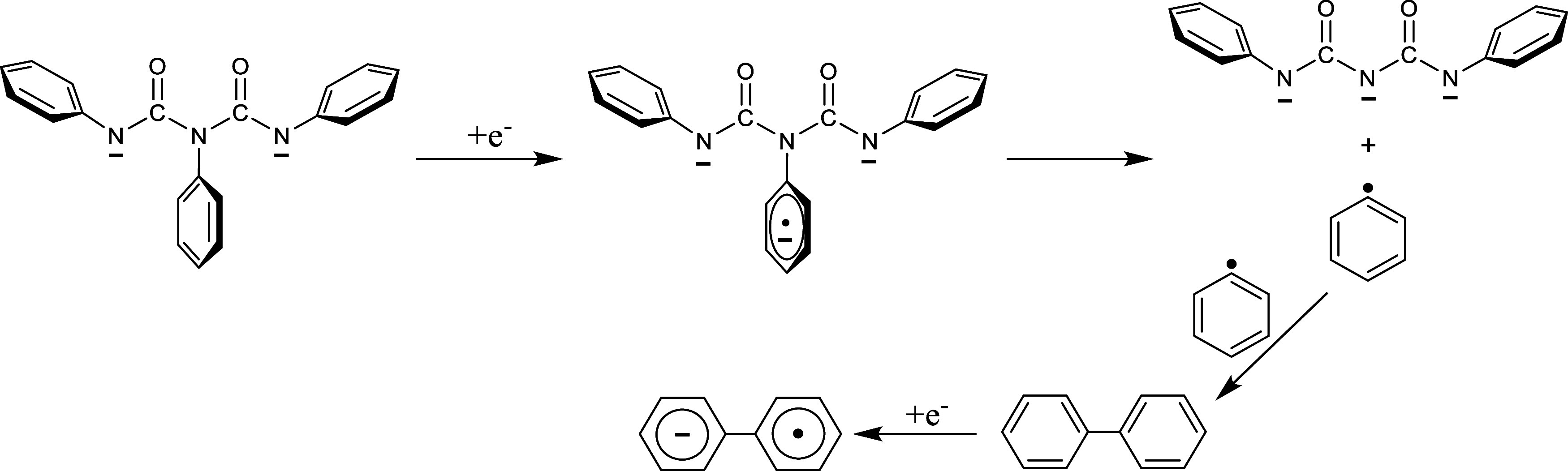
Proposed Reaction Pathway for Formation of the Biphenyl Anion Radical after Further Reduction of the Triphenylbiuret Dianion. The Same Reaction Applies to All Triarylbiuret Dianion Systems (Aryl = Phenyl, *p*-Tolyl, 4′-Biphenylyl, 3,5-Dimethylphenyl, or 1′-Naphthyl)

**Figure 9:**
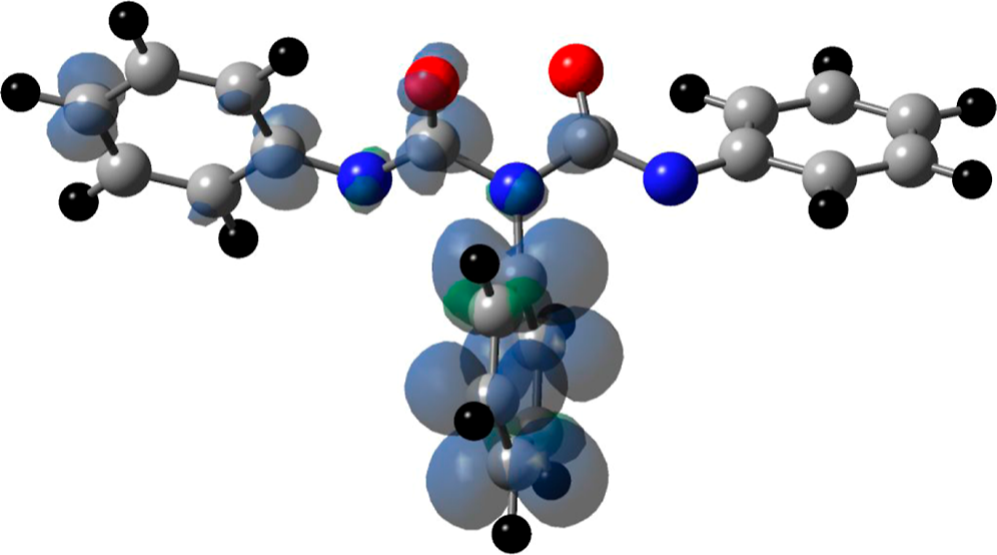
UB3LYP/6-31+G(d,p) optimized geometry for N, *N*′,*N*″-triphenylbiuret trianion radical (**2d**^•3–^). The unpaired electron spin density map is also displayed as a contour plot. The transparent blue and green colors represent positive and negative spin densities. Atom colors: carbon (gray), nitrogen (blue), oxygen (red), and hydrogen (black).

The EPR results suggest that continued addition of more metal to the solution that contains the newly formed triarylbiuret dianions (e.g., **1d**^2–^ – 5d^2–^) leads to the formation of the biaryl anion radicals (e.g., **1c**^•–^ – **4c**^•–^) and the perylene anion radical (**5c**^•–^). Therefore, we propose that continued reduction of the triarylbiuret dianion generates an unstable trianion radical that subsequently undergoes a bond cleavage reaction to release a reactive aryl radical. This joining of two aryl radicals would produce biaryls. Scheme 2 depicts this reaction mechanism for the one-electron reduction of **2d**^2–^ and the fragmentation that follows for the triphenylbiuret trianion radical (**2d**^•3–^). The heterolytic bond cleavage of the middle aromatic C–N σ bond in **2d**^•3–^ releases a phenyl radical that reacts intermolecularly with a second phenyl radical to produce biphenyl, which is then reduced to the anion radical. As mentioned earlier, multiple electron reductions have been shown to cause heterolytic bond cleavage in the reduction of triaryl amines and other arene systems, leading to the formation of biaryls.^23−26^ The proposed formation of aryl radicals may account for the low biaryl yields obtained in these reduction experiments, since it is likely these radical intermediates will undergo additional chemistry with other species in solution. The choice of which aryl C–N bond undergoes heterolytic cleavage was determined from DFT computations of **2d**^•3–^ using the same protocol as that described in the optimization of **2d**^2–^. The optimized geometry for **2d**^•3–^ is shown in Figure 9 along with the calculated spin density map for the unpaired electron. We find that the unpaired electron is predominately localized in the π system of the central phenyl ring, suggesting that this is the ring that is involved in the heterolytic bond cleavage. The release of aryl radicals has been proposed in anion radicals of diarylfluorenes and of aromatic ethers, where it was suggested that excess electron density is considered to reside in the region of high spin density within the aryl group fragment.^34,35^ We were unable to obtain experimental evidence to support breakage of the central C–N bond; therefore, we cannot rule out the possibility that one of the other two aryl C–N bonds may also undergo fragmentation.

**Figure 10:**
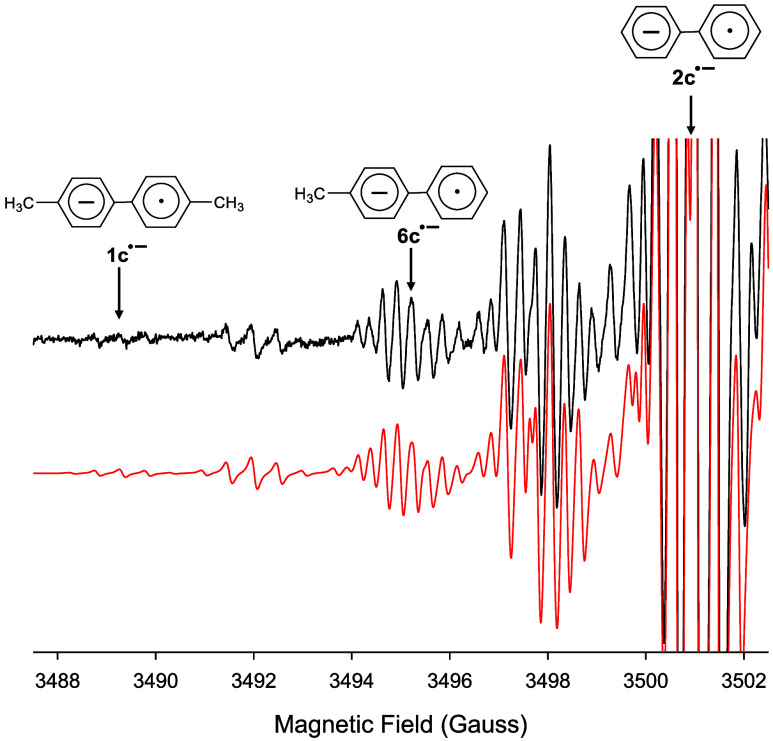
(Black) EPR spectrum showing only the first 15 G recorded at 295 K after addition of *K* metal to a THF solution containing a 5:1 ratio of triphenyl- and tri*p*-tolyl- isocyanurates and a molar excess of 18-crown-6 under vacuum. The full EPR spectrum is shown in Figure S6. (Red) Computer generated simulation using the same a_H_’s given in Figure 2 and in Figure S2. Additional a_H_’s of 2.79, 2.49, 0.59, and 0.30 G for four sets of 2H atoms, 5.59 G for 3H atoms, and 5.39 G for a single H atom was included and Δ*w*_pp_ = 0.13 G. A ratio of 0.2:4.8:1.0 for **1c**^•–^: **2c**^•–^: **6c**^•–^ was used to generate the simulation. Note that the first resonances associated with **6c**^•–^ overlap with those for **1c**^•–^. The first pentet of resonances for **2c**^•–^ is off scale. The vertical arrows mark the center of the first downfield resonances for all three anion radicals.

The intermolecular joining of two aryl radicals would suggest that nonsymmetric biaryls (i.e., two different aryl rings bonded together) could be generated when different triaryl isocyanurates are reduced together in the same solution. We carried out such an experiment with a 5:1 mixture of **2b**/**1b**, respectively, using the same THF/18-crown-6 conditions described above. Upon reduction of the solution with enough *K* metal, EPR analysis revealed that the biaryl anion radical of 4-methylbiphenyl (**6c**^•–^) was indeed generated. Also produced in the reduction was **2c**^•–^ as the major product and **1c**^•–^ as the minor product, Figure 10. The coupling constants for **6c**^•–^ were obtained in a separate experiment by reducing authentic **6c** with *K* metal in a THF solution containing 18-crown-6, see Figure S7. The ratio of the three combined spectra used to generate the red simulation in Figure 10 is very close to the predicted percentages of all three anion radicals in solution, assuming a random joining of the two aryl rings and the initial 5:1 ratio of **2b** and **1b**. Formation of **6c**^•–^ is strong evidence that the biaryls are generated from an intermolecular joining of two aryl rings upon breakdown of the triarylbiuret trianion radical.

## Conclusions

Multiple electron reductions of aryl isocyanates are shown to lead to the formation of biaryl anion radicals (see Figure 5). This is achieved by the potassium metal reductions in THF with excess 18-crown-6 or in HMPA under vacuum conditions at room temperature. The overall reaction mechanism proposed involves the initial formation of the triaryl isocyanurate anion radical from the analogous isocyanate early in the reduction process. Continued exposure to metal further reduces the triaryl isocyanurate to the dianion, which undergoes elimination of CO to generate a triarylbiuret dianion. The biuret dianion formation is supported by an independent reduction experiment using authentic triphenyl isocyanurate, which was monitored using NMR spectroscopy. Further addition of electrons (e.g., exposure to more metal) to the solution containing this biuret dianion leads to formation of an unstable trianion radical, which undergoes a reductive bond cleavage with the loss of an aryl radical. An intermolecular bonding of two aryl radicals results in formation of biaryls, followed by their one-electron reduction to the respective anion radicals. The same outcome occurs when the reduction process is performed with triaryl isocyanurates. We demonstrate that the reduction of a mixture of different triaryl isocyanurates in the same solution will generate mixed biaryl anion radicals. The experiments described should be applicable to the formation and observation of a wide variety of biaryl and mixed biaryl anion radicals formed in one procedural method starting from the aryl isocyanates, and therefore the reduction of a host of substituted aryl isocyanates awaits empirical study.

## Experimental Section

### Reduction of Aryl Isocyanate Compounds in HMPA and THF

A sealed glass tube (with fragile ends) was charged with 0.22 mmol of aryl isocyanate and placed into bulb E of a Pyrex glass apparatus, as shown in Figure S8. A small amount of potassium metal was placed into bulb B, which was then sealed at point A. With experiments using THF as the solvent, 0.27 mmol of 18-crown-6 was also placed in bulb E directly. To ensure that the crown ether was dry, the apparatus was evacuated and left open to the vacuum pump overnight. Next, the *K* metal was distilled into bulb D to form a pristine metal mirror, and then bulb B was removed at point C. Approximately, 2.5 mL of HMPA (dried with potassium metal) or THF (dried over NaK) was distilled directly into bulb E while the system remained under vacuum. The apparatus was sealed and removed from the vacuum line. The glass tube containing the aryl isocyanate was opened, and the solution was well mixed before beginning the exposure to the *K* metal mirror. Once the reduction process began, a portion of the solution was added to the 3 mm EPR tube and analyzed with an X-band Bruker spectrometer operating with Xenon software and equipped with a variable temperature unit. Data was collected with the following EPR parameters: microwave power = 23 dB, modulation amplitude = 0.15 G, time constant = 0.01 ms, and sampling time = 293 ms. The apparatus can be removed from the EPR instrument, and the solution re-exposed to more metal and reanalyzed with the EPR instrument. This process was continued until the solution was significantly reduced with metal, and the signal for the anion radicals detected was optimized. The same procedure was used for the reduction of the triphenyl- and tri*p*-tolyl isocyanurate compounds. The reduction of these compounds was carried out using a 4:1 ratio of 18-crown-6 to triaryl isocyanurate when performed in THF or in HMPA with no crown ether present.

The NMR experiments involving the reduction of triphenyl isocyanurate were performed in a THF-*d*_8_ solution containing a 3× molar excess of 18-crown-6 using a procedure similar to that described above. The glass apparatus was modified to include a side arm with three 5 mm NMR tubes attached. Aliquots of the THF-*d*_8_ solution were placed in the NMR tubes as the reduction with K metal was carried out. The shade of orange color was a qualitative measure for determining when to harvest the NMR samples for analysis. In addition to the 1D NMR data collected, structural assignments were made with additional information from gCOSY, gHSQC, and gHMBC experiments.

#### Safety Statement

Caution! Caution should be exercised when using isocyanates. Organic isocyanates are flammable and corrosive, can cause severe skin burns, and are a respiratory irritant. Caution! Care should be taken when handling HMPA, which causes severe skin burns and may cause genetic defects and cancer. Caution! Care should be taken when handling THF, which is a highly flammable liquid and respiratory irritant and suspected of causing cancer. Caution! Care should be taken when working with potassium metal and avoid exposure to water. The metal is flammable and can cause severe burns. Caution! Extreme care should be taken in handling cryogen liquid nitrogen and its use in vacuum line traps to avoid the condensation of oxygen from air.

#### Computational Methodology

Geometry optimizations for the triphenyl biuret dianion (**2d**^2–^) and the trianion radical (**2d**^•3–^) structures were carried out via DFT calculations using the B3LYP/6-31+G(d,p) basis set with scrf = (cpcm, solvent = THF, ε = 7.42). Isotropic NMR shielding tensors (ppm) were computed for **2d**^2–^ and TMS with the gauge-independent atomic orbital method from the optimized geometries at the same level of theory. The computed carbon and hydrogen shielding tensors (σ_C,calc_ and σ_H,calc_) for **2d**^2–^ were referenced using the carbon and hydrogen tensors for TMS. For example, σ_C,calc_ = σ_C,TMS_ – σ_C,target_ where σ_C,target_ represents the average of all computed shielding tensor for equivalent carbons in **2d**^2–^. The same expression can be used for calculating the hydrogen shielding tenors, σ_H,calc_. The target shielding tenor values can be found in Supporting Information. All calculations were implemented in Gaussian 16, Revision C.01.^45^

## Data Availability

The data underlying this study are available in the published article and its Supporting Information.

## Supporting Information Available

Additional EPR spectra for reduction studies on aryl isocyanates, ^13^C-{^1^H}-NMR and 2D NMR spectral data, and DFT calculations including computed shielding tensor values for **2d**^2–^ and TMS (PDF)
