# Supplementary material for: Biaryl Anion Radical Formation by Potassium Metal Reduction of Aryl Isocyanates and Triaryl Isocyanurates
Source: J Org Chem. 2024 Oct 13;89(21):15708–17. doi: 10.1021/acs.joc.4c01844 (PMC11536357; doi:10.1021/acs.joc.4c01844)

Supporting Information

Biaryl Anion Radical Formation by Potassium Metal Reduction of Aryl Isocyanates and Triaryl Isocyanurates

Steven J. Peters,* Sean H. Kennedy, and Colton J. Christiansen

Department of Chemistry, Illinois State University, Normal, Illinois 61790-4160

sjpeter@illinoisstate.edu

Table of Contents **Page No.**

Figure S1. Sample tubes containing THF solutions collected from the sequential potassium metal reduction of a THF solution containing *p*-tolyl isocyanate (**1a**) and excess 18-crown-6. The extent of the reduction with metal increases from left to right for each sample shown. S3

Figure S2. X-band EPR spectra of biphenyl anion radical formed from reduction of phenyl isocyanate in THF with excess 18-crown-6. S3

Figure S3. X-band EPR spectra of 3, 3', 5, 5'-tetramethyl biphenyl anion radical formed from reduction of 3,5-dimethylphenyl isocyanate in THF with excess 18-crown-6. S4

Figure S4. 400 MHz ^{1}H-^{1}H-COSY NMR spectrum of a THF-d_8 solution containing tri-phenyl isocyanurate (**2b**) with two equivalence of 18-crown-6 reduced with K metal. S5

Figure S5. 400 MHz-100 MHz ^{13}C-^{1}H-HSQC (red) and HMBC (black) NMR spectra overlayed. These spectra are of a THF-d_8 solution containing tri-phenyl isocyanurate (**2b**) with two equivalence of 18-crown-6 reduced with K metal under vacuum. S6

Figure S6. Full EPR spectrum of a solution containing a 5:1 ratio of tri-phenyl- and tri-*p*-tolyl- isocyanurates reduced with K metal in THF/18-crown-6. S7

Figure S7. X-band EPR spectrum recorded at 295 K after addition potassium metal to a THF solution containing 4-methylbiphenyl and 18-crown-6 under vacuum. S8

Figure S8. Glass apparatus used in the EPR experiments for the potassium metal reduction of aryl isocyanate. S9

B3LYP/6-31+G(d,p) computational information for triphenyl biuret dianion and trianion radical. The computed isotropic shielding tensors for the dianion and TMS are included. S10-S14

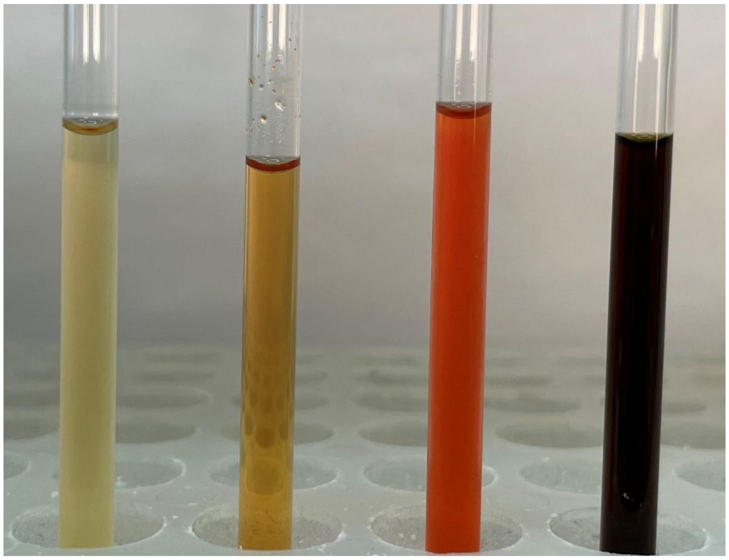

Figure S1. Sample tubes containing THF solutions collected from the sequential potassium metal reduction of a THF solution containing *p*-tolyl isocyanate (**1a**) and excess 18-crown-6. The extent of the reduction with metal increases from left to right for each sample shown.

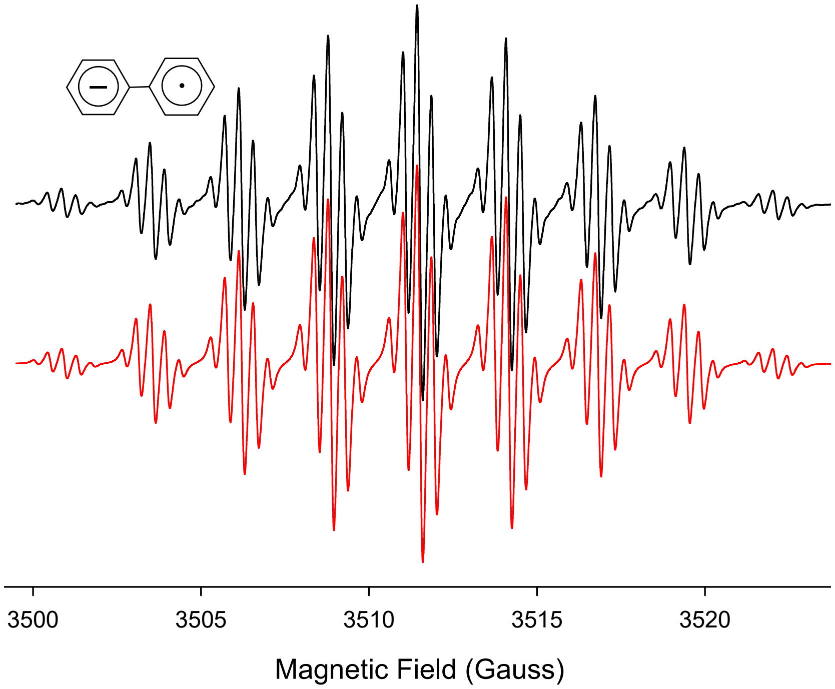

Figure S2. (Black) X-band EPR spectrum recorded at 295 K after addition potassium metal to a THF solution containing phenyl isocyanate (**2a**) and 18-crown-6 ([18-crown-6] = 2 x [**2a**]) under vacuum. (Red) Computer-generated simulation using a_H's of 0.42 G and 2.63 G for two sets of 4 H atoms, and a_H of 5.32 G for 2 H atoms, Δw_{pp} = 0.17 G.

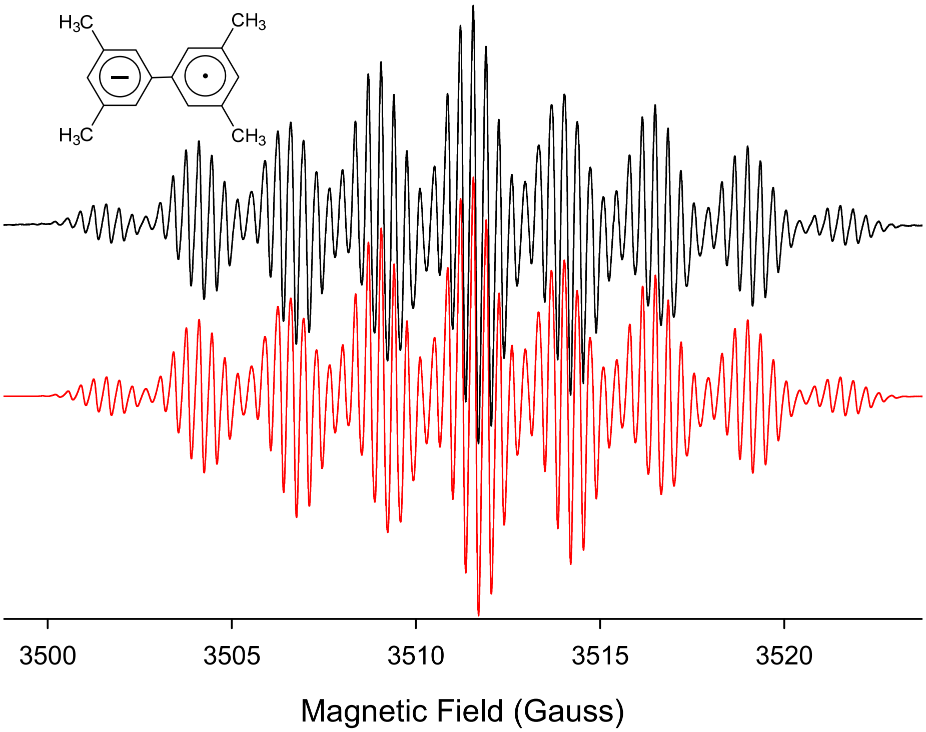

Figure S3. (Black) X-band EPR spectrum recorded at 295 K after addition potassium metal to a THF solution containing 3,5-dimethylphenyl isocyanate (**3a**) and 18-crown-6 ([18-crown-6] = 2 x [**3a**]) under vacuum. (Red) Computer-generated simulation using a_H's of 0.35 G for 12 H atoms, 2.52 G for 4 H atoms, and a_H of 4.93 G for 2 H atoms, Δw_{pp} = 0.14 G.

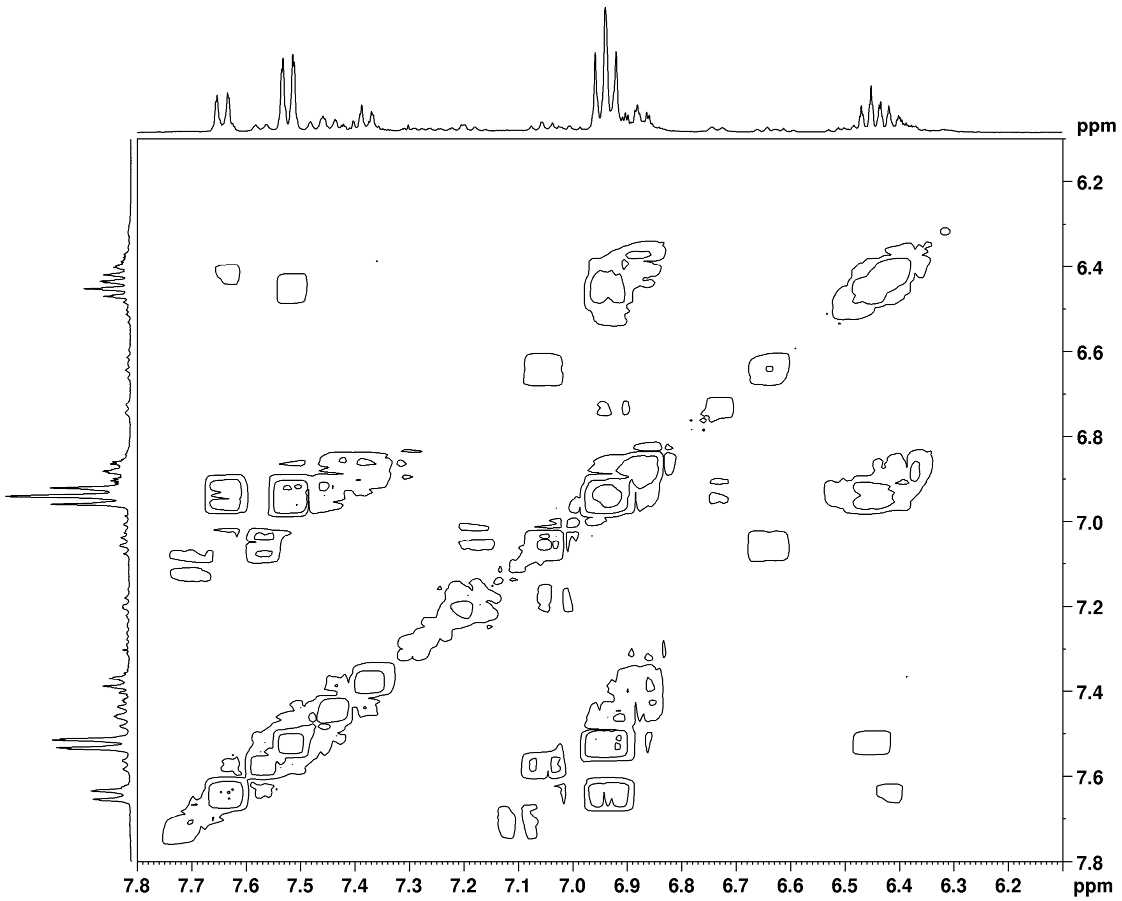

Figure S4. 400 MHz ^{1}H-^{1}H-COSY NMR spectrum of a THF-d_8 solution containing tri-phenyl isocyanurate (**2b**) with two equivalence of 18-crown-6 reduced with K metal under vacuum. This spectrum was collected after the third NMR sample was collected and the solution was extensively reduced where [K] >> [**2b**].

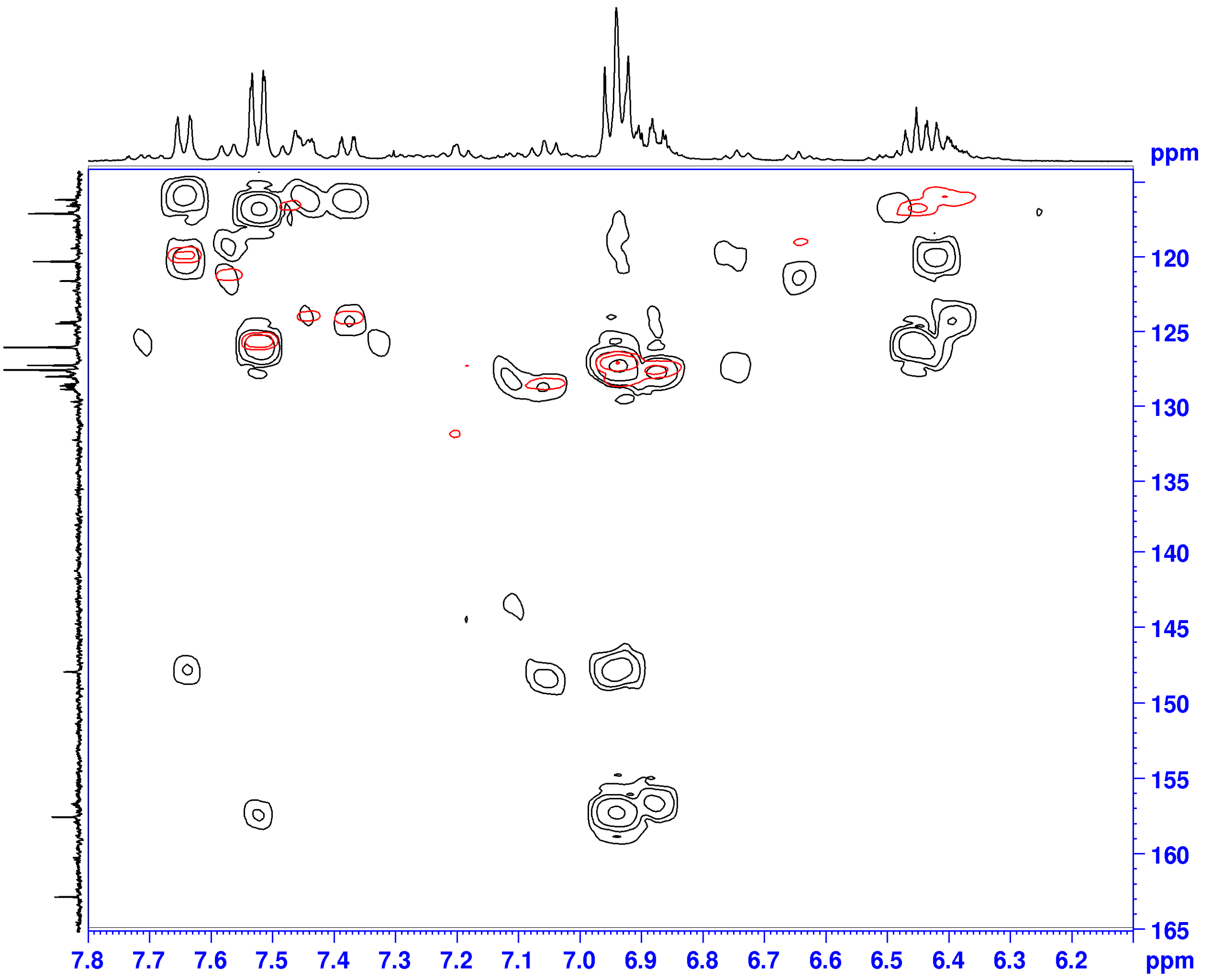

Figure S5. 400 MHz-100 MHz ^{13}C-^{1}H-HSQC (red) and HMBC (black) NMR spectra overlayed. These spectra are of a THF-d_8 solution containing tri-phenyl isocyanurate (**2b**) with two equivalence of 18-crown-6 reduced with K metal under vacuum and were collected after the third NMR sample was harvested once the solution was extensively reduced ([K] >> [**2b**]). The $^{3}J_{CH}$ correlations are considerable stronger than the $^{2}J_{CH}$ correlations.

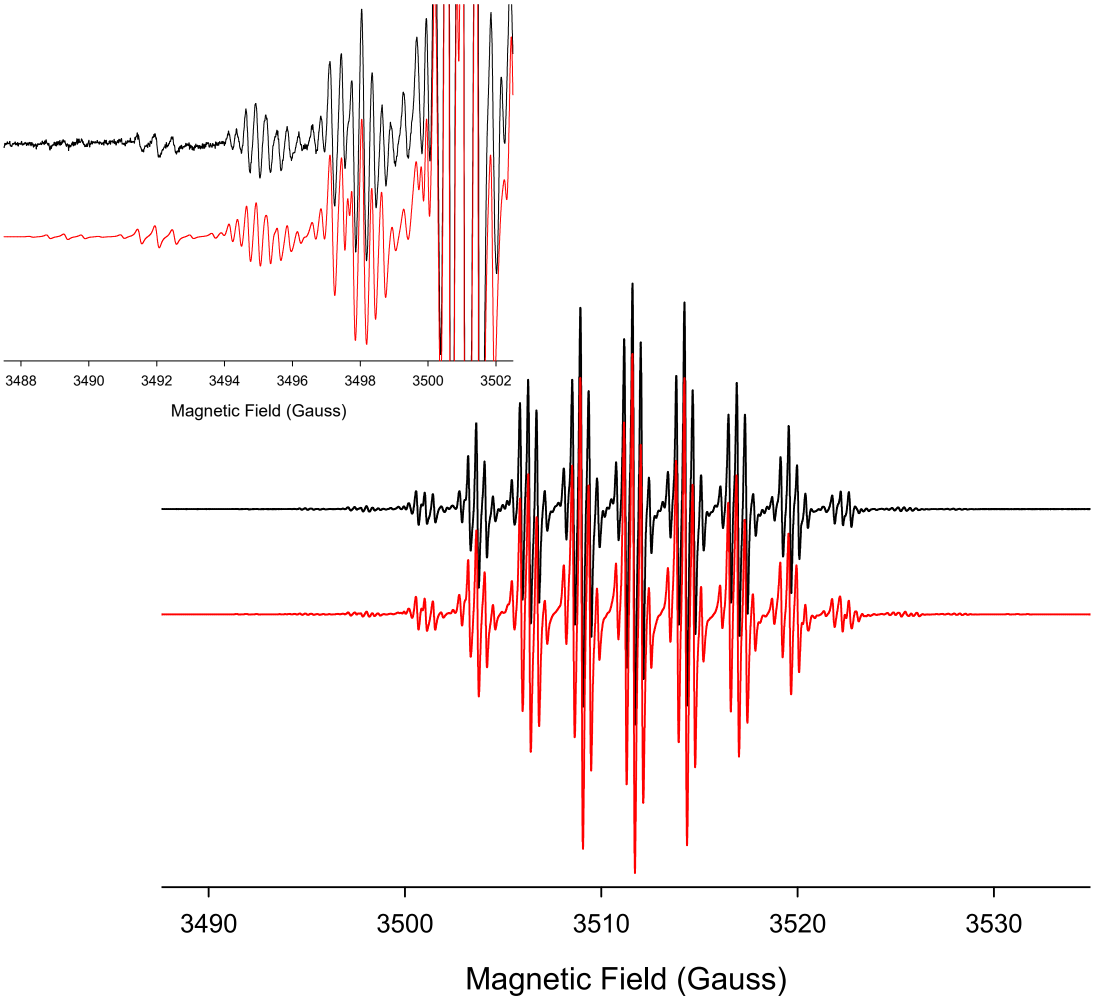

Figure S6. (Black) EPR spectrum recorded at 295 K after a THF solution containing a 5:1 ratio of tri-phenyl- and tri-*p*-tolyl- isocyanurates with a molar excess of 18-crown-6 addition is reduced with K metal to under vacuum. (Red) Computer generated simulation using the same a_H's given in Figure 2 for **1c**$^{\bullet-}$and in Figure S1 for **2c**$^{\bullet-}$. Additional a_H's of 2.79 G, 2.49 G 0.59 G and 0.30 G for four sets of 2 H atoms, 5.59 G for 3 H atoms and 5.39 G for a single H atom for **6c**$^{\bullet-}$ was included and Δw_{pp} = 0.13 G. A ratio of 0.2 : 4.8 : 1.0 for **1c**$^{\bullet-}$: **2c**$^{\bullet-}$: **6c**$^{\bullet-}$ was used to generate the simulation. Note that the first resonances associated with **6c**$^{\bullet-}$ overlaps with those for **1c**$^{\bullet-}$. The inset shows only the first 15 Gauss of the spectrum.

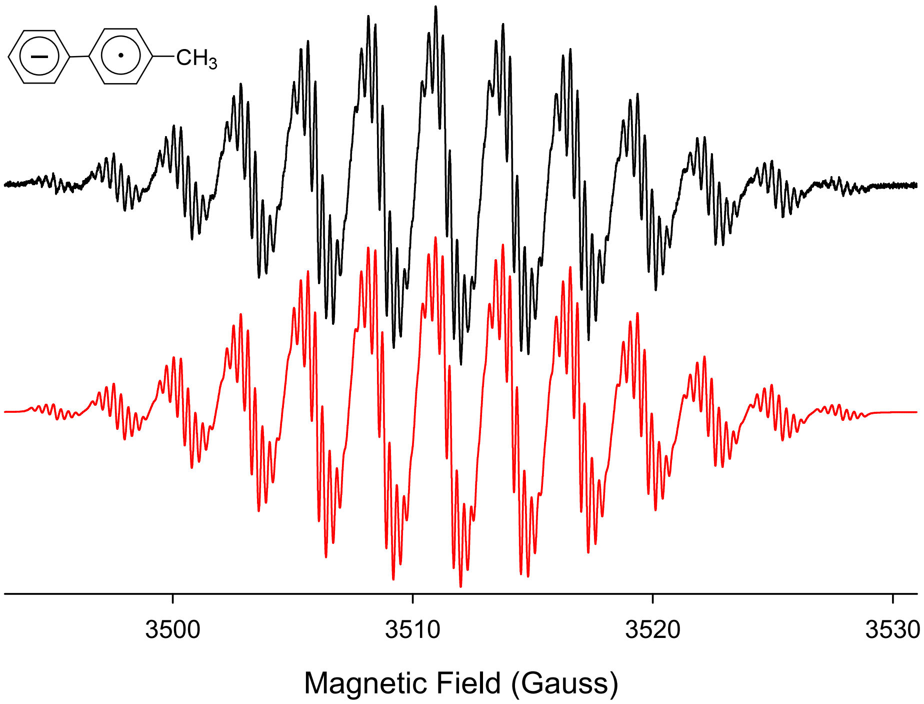

Figure S7. (Black) X-band EPR spectrum recorded at 295 K after addition potassium metal to a THF solution containing 4-methylbiphenyl and 18-crown-6 under vacuum. (Red) Computer simulation using a_H's of 2.52 G, 2.79 G, 0.61 G and 0.30 G for four sets of 2 H atoms, a_H of 5.53 G for 3 H atoms, and 5.38 H for a single H atom, Δw_{pp} = 0.19 G.

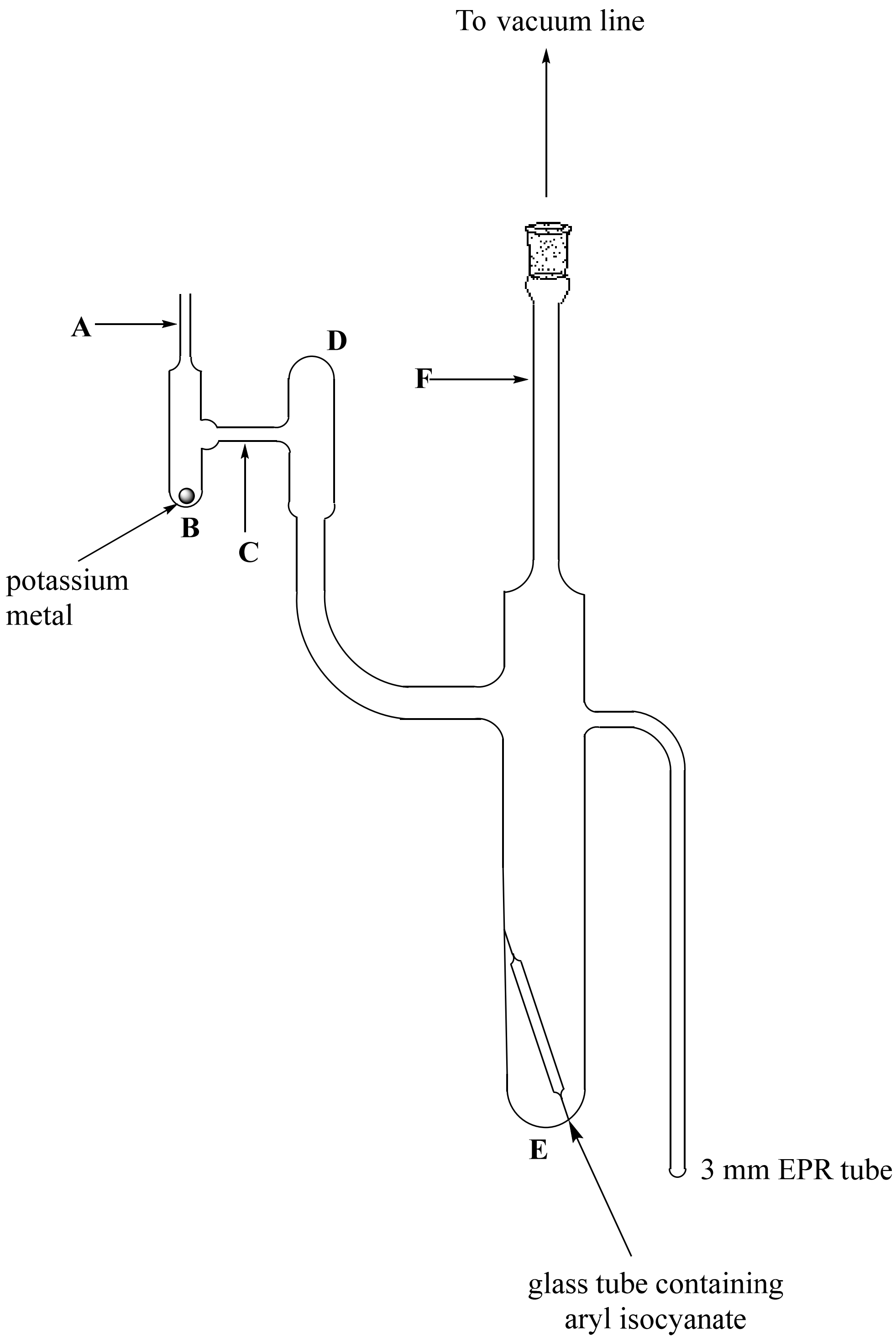

Figure S8. Glass apparatus used in the EPR experiments for the potassium metal reduction of aryl isocyanate.

Triphenylbiuret dianion computational results

XYZ Coordinates

Center Number	Atomic Number	Coordinates (Angstroms) X	Y	Z
1	7	2.279840	-0.186886	-0.415962
2	6	1.227428	-0.715467	0.218516
3	7	-0.000064	0.025261	-0.000080
4	6	-1.227332	-0.715812	-0.217694
5	7	-2.280144	-0.186074	0.415226
6	6	3.572970	-0.629062	-0.198786
7	6	6.348908	-1.303053	0.067260
8	6	4.025755	-1.566078	0.775233
9	6	4.577043	-0.055442	-1.029668
10	6	5.924519	-0.383539	-0.904053
11	6	5.382601	-1.880676	0.899181
12	1	3.294999	-2.038018	1.417813
13	1	4.257591	0.664043	-1.779686
14	1	6.651329	0.082657	-1.566326
15	1	5.686933	-2.598013	1.659578
16	1	7.399483	-1.561610	0.168902
17	6	-0.000067	1.433182	-0.000005
18	6	0.000133	4.267251	-0.000031
19	6	-0.777777	2.158718	-0.923062
20	6	0.777734	2.158624	0.923038
21	6	0.782487	3.554615	0.916750
22	6	-0.782330	3.554695	-0.916803
23	1	-1.381486	1.618229	-1.644788
24	1	1.381372	1.618096	1.644801
25	1	1.393261	4.087721	1.641310
26	1	-1.393015	4.087878	-1.641382
27	1	0.000210	5.353655	-0.000050
28	6	-3.573128	-0.628663	0.198197
29	6	-6.348923	-1.303393	-0.067798
30	6	-4.025513	-1.567046	-0.774709
31	6	-4.577546	-0.054113	1.028036
32	6	-5.924939	-0.382563	0.902447
33	6	-5.382276	-1.881984	-0.898657
34	1	-3.294503	-2.039751	-1.416436
35	1	-4.258424	0.666363	1.777245
36	1	-6.652005	0.084391	1.563904
37	1	-5.686278	-2.600359	-1.658206
38	1	-7.399433	-1.562224	-0.169416
39	8	1.157663	-1.735941	0.950302
40	8	-1.157154	-1.737849	-0.947291

E(RB3LYP) = -1086.18470093 Hartree

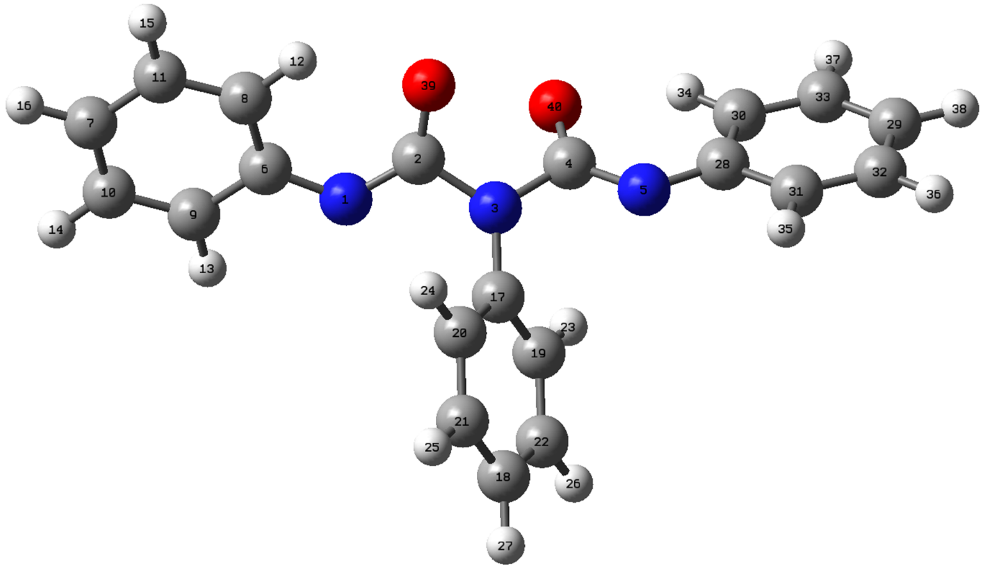

B3LYP/6-31+G(d, p) scrf=(cpcm,solvent=thf)

SCF GIAO Magnetic shielding tensor (ppm):

1 N Isotropic = 69.5723
2 C Isotropic = 29.8427
3 N Isotropic = 87.4987
4 C Isotropic = 29.8510
5 N Isotropic = 69.6176
6 C Isotropic = 37.5877
7 C Isotropic = 82.8649
8 C Isotropic = 75.2427
9 C Isotropic = 71.0278
10 C Isotropic = 69.3513
11 C Isotropic = 68.5505
12 H Isotropic = 22.6638
13 H Isotropic = 24.9651
14 H Isotropic = 24.4846
15 H Isotropic = 24.3615
16 H Isotropic = 25.0681
17 C Isotropic = 44.2144
18 C Isotropic = 78.5187
19 C Isotropic = 70.4337
20 C Isotropic = 70.4564
21 C Isotropic = 69.7955
22 C Isotropic = 69.7998
23 H Isotropic = 24.5950
24 H Isotropic = 24.5962
25 H Isotropic = 24.4251
26 H Isotropic = 24.4249

27 H Isotropic = 24.8226
28 C Isotropic = 37.5826
29 C Isotropic = 82.8742
30 C Isotropic = 75.2377
31 C Isotropic = 71.0331
32 C Isotropic = 69.3489
33 C Isotropic = 68.5529
34 H Isotropic = 22.6656
35 H Isotropic = 24.9656
36 H Isotropic = 24.4850
37 H Isotropic = 24.3621
38 H Isotropic = 25.0689
39 O Isotropic = 31.7778
40 O Isotropic = 31.9466

Tetramethylsilane (TMS) SCF GIAO Magnetic shielding tensor (ppm):

2 C Isotropic = 193.1241
3 C Isotropic = 193.1128
4 C Isotropic = 193.1229
5 C Isotropic = 193.1201
6 H Isotropic = 31.6387
7 H Isotropic = 31.6393
8 H Isotropic = 31.6393
9 H Isotropic = 31.6402
10 H Isotropic = 31.6390
11 H Isotropic = 31.6374
12 H Isotropic = 31.6386
13 H Isotropic = 31.6404
14 H Isotropic = 31.6378
15 H Isotropic = 31.6397
16 H Isotropic = 31.6400
17 H Isotropic = 31.6396

Triphenylbiuret trianion radical computational results

B3LYP/6-31+G(d, p) scrf=(cpcm,solvent=thf)

Center Number	Atomic Number	Coordinates (Angstroms) X	Y	Z
1	7	2.363928	-0.074797	-0.510956
2	6	1.201480	-0.733819	-0.289234
3	7	0.048811	0.074348	-0.343775
4	6	-1.245841	-0.530347	-0.617818
5	7	-2.151869	-0.312126	0.349844
6	6	3.576214	-0.613141	-0.165816
7	6	6.243582	-1.466148	0.517999
8	6	3.821187	-1.644390	0.796268
9	6	4.741909	-0.045096	-0.766163
10	6	6.030494	-0.458997	-0.437534
11	6	5.117500	-2.043120	1.125058
12	1	2.972015	-2.122273	1.268473
13	1	4.593712	0.744820	-1.499348
14	1	6.880912	0.011177	-0.929050
15	1	5.251665	-2.825819	1.870964
16	1	7.248166	-1.790054	0.777046
17	6	0.069869	1.483354	-0.152647
18	6	-0.051237	4.321186	0.285676
19	6	-0.458574	2.356215	-1.132436
20	6	0.511204	2.072111	1.093640
21	6	0.449753	3.448538	1.280231
22	6	-0.535106	3.733846	-0.937380
23	1	-0.820121	1.927000	-2.066290
24	1	0.904627	1.426634	1.874998
25	1	0.799024	3.865189	2.226317
26	1	-0.941813	4.365013	-1.726125
27	1	-0.101600	5.393887	0.454167
28	6	-3.471025	-0.691819	0.247855
29	6	-6.275701	-1.377060	0.332465
30	6	-4.140250	-1.300285	-0.866673
31	6	-4.287500	-0.465066	1.404290
32	6	-5.636866	-0.795374	1.446202
33	6	-5.497390	-1.622220	-0.812458
34	1	-3.562713	-1.505850	-1.757967
35	1	-3.810960	-0.009983	2.270179
36	1	-6.204808	-0.595358	2.353875
37	1	-5.960900	-2.077344	-1.687628
38	1	-7.331652	-1.633047	0.360558
39	8	1.057213	-1.972800	-0.066140

40	8	-1.397694	-1.121721	-1.729500

E(UB3LYP) = -1086.17773821 Hartree

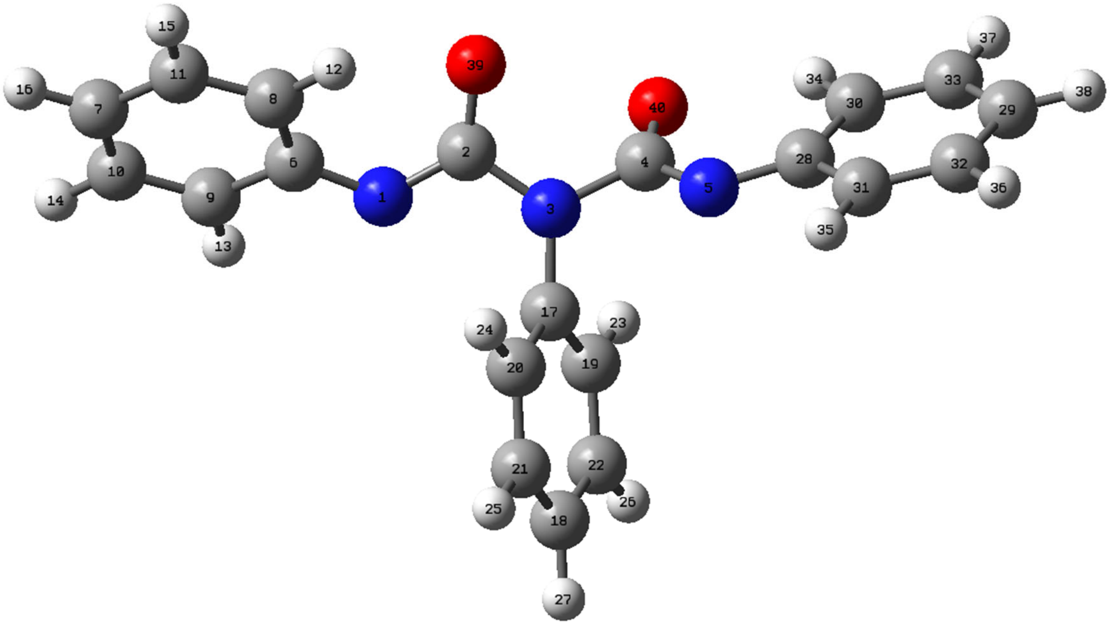

Supplement: Supplementary file 1 — jo4c01844_si_001.pdf [file jo4c01844_si_001.pdf]
